# Coffee and tea intake in relation to subjective cognitive decline: A cross‐sectional study

**DOI:** 10.1002/alz70860_101461

**Published:** 2025-12-23

**Authors:** Minqing Yan, Zifan Lin, Jinghong Lin, Yuqi Yang, Dong Zhao, Mengjie He, Ting Shen, Gulisiya Hailili, Liyan Huang, Yiying Gong, Ronghua Zhang, Changzheng Yuan

**Affiliations:** ^1^ Zhejiang University, Hangzhou, Zhejiang, China; ^2^ Zhejiang University, Hangzhou, Zhejiang province, China; ^3^ Zhejiang Provincial Center for Disease Control and Prevention, Hangzhou, Zhejiang province, China; ^4^ Harvard T. H. Chan School of Public Health, Boston, MA, USA

## Abstract

**Background:**

Evidence regarding the role of coffee and tea intake in subjective cognitive decline (SCD) remains limited and inconclusive.

**Method:**

We conducted a cross‐sectional analysis among 3,476 schoolteachers aged 45 years and above (mean age 50.1 ± 3.7 years, 62.0% female) in China. Coffee and tea intake was assessed using a 36‐item simplified Food Frequency Questionnaire (FFQ). Self‐reported SCD was ascertained by a 7‐item questionnaire on memory and cognition changes. Categorical SCD score was classified as “none” (0 points), “moderate” (1‐4 points), and “severe” (5‐7 points). Logistic regression models were used to evaluate the corresponding associations.

**Result:**

Among the study participants, 1,752 (50.4%) participants were classified with moderate SCD, and 847 (24.4%) with severe SCD. The multivariable‐adjusted odds ratio (OR) for moderate SCD among coffee drinkers compared to non‐drinkers was 0.80 (95% confidence interval [CI]: 0.67, 0.96). Participants who drank tea >3 times/week also had lower odds of severe SCD compared to none consumers (OR = 0.66, 95% CI, 0.47, 0.92). Generally, no significant interactions were found between coffee and tea intake and major covariates. However, a stronger association between tea intake and SCD was observed among younger participants <50 years old (*p* interaction < 0.001 for moderate SCD, *p* interaction = 0.011 for severe SCD).

**Conclusion:**

Our findings support the potential beneficial roles of coffee and tea for the maintenance of subjective cognitive function among Chinese schoolteachers. Further large‐scale prospective and interventional studies are warranted to confirm these findings.

